# You Are What You Tweet: Connecting the Geographic Variation in America’s Obesity Rate to Twitter Content

**DOI:** 10.1371/journal.pone.0133505

**Published:** 2015-09-02

**Authors:** Ross Joseph Gore, Saikou Diallo, Jose Padilla

**Affiliations:** Virginia Modeling, Analysis and Simulation Center, Old Dominion University, Norfolk, VA, United States of America; McMaster University, CANADA

## Abstract

We conduct a detailed investigation of the relationship among the obesity rate of urban areas and expressions of happiness, diet and physical activity on social media. We do so by analyzing a massive, geo-tagged data set comprising over 200 million words generated over the course of 2012 and 2013 on the social network service Twitter. Among many results, we show that areas with lower obesity rates: (1) have happier tweets and frequently discuss (2) food, particularly fruits and vegetables, and (3) physical activities of any intensity. Additionally, we provide evidence that each of these results offer different and unique insight into the variation of the obesity rate in urban areas within the United States. Our work shows how the contents of social media may potentially be used to estimate real-time, population-scale measures of factors related to obesity.

## Introduction

Obesity is becoming increasingly problematic and common in the United States population [1, 2]. More than one-third of U.S. adults are obese resulting in an annual medical cost of over $150 billion dollars [1, 3, 4]. These medical costs occur because obese people are significantly more prone to the leading causes of preventable death including: heart disease, stroke and type 2 diabetes [5]. Obesity is defined by a Body-Mass Index (BMI) which reflects an individual’s weight divided by square of their height. Obese individuals have a BMI of 30 kg m^2^ or greater. Obesity rate is defined as the percentage of the people in a Metropolitan Statistical Area (MSA) who have a BMI of 30 kg m^2^ or greater [2, 6].

Despite the prevalence of obesity in the U.S. it is not problematic to the same degree across the country. According to the 2012–2013 Gallup-Healthways Wellness Survey (GHWS) the obesity rate of U.S. MSAs ranges from 12.4% (Boulder, CO) to 39.5% (Huntington, WV). The lack of uniformity in the obesity rate has motivated researchers to identify the factors that can affect obesity and offer insight into the variation in the data [7].

While the GHWS and other approaches to quantifying the well being of a city rely almost exclusively on survey data, there are now a range of complementary, remote-sensing methods available to researchers. The explosion in the amount and availability of data relating to social media in the past 10 years has driven a rapid increase in the application of data-driven techniques to the social sciences and other analyses of large-scale populations.

Our overall aim in this paper is to investigate how the obesity rate of an urban geographic area correlates with the contents of geo-tagged tweets in that area. Here, tweets refer to 140 character microblogs expressed on the social media platform www.twitter.com and urban areas reflect the 189 MSAs defined by the U.S. Office of Management and Budget [8]. In particular we ask four research questions using geo-tagged tweets from 2012–2013:

1. How is the average happiness of the tweets in an urban area related to the population’s obesity rate?
2. How is the overall discussion of food consumption on Twitter, and the nutritional density of the food discussed, in related an urban area related to the population’s obesity rate?
3. How is the overall discussion of physical activity on Twitter, and the intensity of the activity discussed, in an urban area related to the population’s obesity rate?
4. To what extent do the measures used to answer these questions offer unique insight and how well does each correlate with a MSA-level survey measure of a similar variable?

Our methodology for answering the first question uses word frequency distributions collected from a large corpus of geo-tagged tweets posted on Twitter, with individual words scored for their happiness independently by users of Amazon’s Mechanical Turk service [9]. This measure was introduced by Dodds and Danforth [10], tested for robustness and sensitivity [11], and employed by Mitchell et. al in a similar pursuit [12].

In answering questions 2 and 3 we explore the extent to which the level of granularity needed to answer the first question is required for the second and third question. To answer the final question we compute the correlations among the measures used to answer the first three questions to gauge how much unique insight they provide. We also evaluate how well each of our derived Twitter measures correlates with a MSA-level survey measure of a similar variable. This analysis helps determine if the measures actually capture the intended variables (happiness, diet and physical activity) as opposed to other unrelated variables.

The answers to these questions are not always intuitive and provide significant insight into the health-related habits of Twitter users in different urban areas. Ultimately, they show how social media may potentially be used to estimate population-scale measures of factors related to obesity.

The remainder of the paper is structured as follows. In the Methods section, we describe the data sets in our study and our measures of happiness, diet and physical activity derived from tweets. In the Results section we demonstrate that obesity rate and happiness have a similar relationship in 2012 and 2013 as the two variables did in 2011. Next, we explore the relationship between the discussion of food consumption on Twitter and the obesity rate in urban areas. Then, we shift our focus to discussions of physical activity. Finally, we explore the extent to which these measures: (1) contain unique insight and (2) match MSA-level survey measures of similar variables. We conclude with a discussion of the validity and limitations of our study along with directions for future work.

## Methods

### Datasets

We examine the relationship between the content of a corpus of geo-tagged tweets (not retweets) and the obesity rate of 189 urban areas in the contiguous United States during the calendar years 2012 and 2013. Our data collection procedure adheres to Twitter’s terms of use/service. It uses Twitter’s streaming API which provides low latency access to Twitter’s global stream of Tweet data. The data we collected reflects a ∼ 10% random sample of all tweets in 2012–2013. From that random sample, 1.5% of the tweets were geo-tagged resulting in a corpus of over 25 million geo-tagged tweets. The geographic boundaries of the urban areas we explore reflect the MSAs defined by U.S. Office of Management and Budget. It is important to note that these urban area boundaries often agglomerate small towns together, particularly when there are small towns geographically close to larger towns or cities.

The obesity rates of the MSAs are provided by the 2012–2013 Gallup Healthways Wellbeing Survey. While other sources of geographic obesity rates exist (i.e. BRFSS and NHANES)[13, 14] we use the GHWS because its data was collected during the same time frame (2012–2013) as our Twitter corpus and (2) it measures other MSA-level variables related to happiness, diet and physical activity which allow us to evaluate additional aspects of our work (i.e. Question 4).

The relationship between these datasets is examined using six measures derived from our Twitter corpus: (a) one related to happiness, (b) three related to diet and (c) two related to physical activity. We define each of these measures next.

### Measure of Happiness

To quantify the happiness of a tweet we employ Mitchell et al.’s measure *h*_*avg*_ which reflects the *happiness* of a tweet. In previous work Mitchell et al. showed that the *happiness* of tweets are correlated with several population-scale measures including household income, education levels and the 2011 obesity rate in MSAs [12].

The *happiness* of a tweet is measured using the Language Assessment by Mechanical Turk (LabMT) word list, assembled by combining the 5,000 most frequent words occurring in each of four text sources: Google Books (English), music lyrics, the New York Times and Twitter. Ten thousand of these individual words have been scored by users of Amazon’s Mechanical Turk service on a scale of 1 (sad) to 9 (happy), resulting in a measure of happiness, *h*, for each given word [9]. For example, ‘rainbow’ is one of the happiest words in the list with a score of 8.10, while ‘earthquake’ is one of the saddest, with a score of 1.90. Neutral words like ‘the’ or ‘thereof’ tend to score in the middle of the scale, with *h*(*the*) = 4.98 and *h*(*thereof*) = 5.00 respectively.

For a given tweet *T* containing *N* unique words the average happiness, *h*_*avg*_. is calculated by:

$$\begin{array}{c} h_{avg}\left(T\right)=\frac{\sum_{i=1}^{N}h\left(w_{i}\right)f_{i}}{\sum_{i=1}^{N}f_{i}}=\sum_{i=1}^{N}h\left(w_{i}\right)p_{i} \end{array}$$ (1)

In Eq 1, *f*_*i*_ is the frequency of the *i*th word *w*_*i*_ in *T* for which we have a happiness value *h*(*w*_*i*_) and $p_{i}=f_{i}/\sum_{i=1}^{N}f_{i}$ is the normalized frequency of the word *w*_*i*_.

### Measures of Diet

To quantify the dietary content of the foods one tweets about we explore three different measures at varying degrees of granularity. Each of these three measures require that we partition our corpus of tweets using the following binary criteria: if a tweet contains a word(s) describing at least one food in the USDA National Nutrient Database (USDANDB) [15] it is placed in the *Food Tweets* set *FT*; otherwise it is placed in the *Non-Food Tweets* set *NFT*.

Given this partitioning, the *Food Tweet %* (*FT*%) of a MSA, is the ratio of *Food Tweets* in the MSA compared to the total number of tweets within the MSA. This reflects our first measure of diet and is shown in Eq 2.

$$\begin{array}{c} FT\%=\frac{\left|FT\right|}{\left(|FT|+|NFT|\right)} \end{array}$$ (2)

While, the *FT*% of a MSA quantifies how frequently people tweet about food, it does not offer any insight into the actual food about which people tweet. To measure how nutritious each food included in each tweet is we measure the average nutrient density, *nd*_*avg*_, of the tweet by using the Nutrient-Rich Foods Index (NRF) formula [16].

While other formulae to determine the nutrient density of foods exist, we use the NRF because its’ scores have been shown to be highly correlated with the recommendations of the USDA’s Healthy Eating Index [17] and diets featuring high nutrient dense foods on the NRF have been been shown to reduce obesity, while diets consisting of low nutrient dense foods increase the prevalence of obesity [18, 19]. Furthermore the NRF is not restricted to any subset of foods. It is generalizable to any food in the USDANDB [20].

Nutrient density in the NRF is determined by computing the daily recommended intake value of protein, dietary fiber, vitamin A, vitamin C, vitamin E, calcium, magnesium, iron and potassium provided per 100 kCals of a given food and then subtracting the daily recommended intake values for saturated fat, sodium and added sugars in 100 kCals of the food. Using this formula, fruits and vegetables are some of the most nutrient dense foods (*nrf*(*spinach*) = 694.8; *nrf*(*strawberries*) = 375.9) while soda is one of the least (*nrf*(*soda*) = −55.8). For a given tweet *T* containing *N* unique foods we calculate the average nutrient density *nd*_*avg*_ using Eq 3.

$$\begin{array}{c} nd_{avg}\left(T\right)=\frac{\sum_{i=1}^{N}nrf\left(food_{i}\right)f_{i}}{\sum_{i=1}^{N}f_{i}}=\sum_{i=1}^{N}nrf\left(food_{i}\right)p_{i} \end{array}$$ (3)

The calculation of *nd*_*avg*_ in Eq 3 is similar to the calculation of *h*_*avg*_. In Eq 3 *f*_*i*_ is the frequency of the *i*th food *food*_*i*_ in *T* with NRF value *nrf*(*food*_*i*_) and $p_{i}=f_{i}\sum_{i=1}^{N}f_{i}$ is the normalized frequency of the food *food*_*i*_. The result is a measure of the average nutrient density of the foods mentioned in a single tweet.

There is a significant difference between the level of granularity in our first measure (*FT*%) and our second (*nd*_*avg*_). To bridge this gap we formulate one more measure of the diet of an MSA: *Produce %* (*Prod*%). *Prod*% marries together the nutritional aspects of *nd*_*avg*_ with the coarse granularity of *FT*%.

Recall, fruits and vegetables are among the most nutritionally dense items on the NRF Index. Any tweet that mentions at least one food listed in either *Fruits and Fruit Juices* or *Vegetable and Vegetable Products* sections of the USDANDB is in set *Prod*. Given this partitioning, *Prod*% is the ratio of tweets in set *Prod* the compared to the total number of tweets in the MSA. This measure is shown in Eq 4.

$$\begin{array}{c} Prod\%=\frac{\left|Prod\right|}{\left(|FT|+|NFT|\right)} \end{array}$$ (4)

### Measures of Physical Activity

Along with happiness and diet, research has shown that the physical activity level of individuals affects obesity [21–23]. With this foundation we explore two different measures to quantify discussions of physical activity within our Twitter data set. Each of these measures require that we partition our corpus of tweets into those that discuss physical activities and those that do not. To do this partition we use a binary criteria similar to our food tweet criteria. If a tweet contains a word(s) discussing at least one physical activity in the guidelines for exercise testing published by the American College of Sports Medicine (ACSM) and the Center for Disease Control and Prevention (CDC) [24] it is placed in the *Physical Activity Tweets* set *PA*; otherwise it is placed in the *Non-Physical Activity Tweets* set *NPA*. While the guidelines for exercise published by the ACSM and CDC are not exhaustive and do not contain every possible physical activity descriptor we employ them in our work because they list over 400 activities and are well established. They been used by the American Heart Association [25], national cross-sectional studies [26] and public health recommendations [27].

Our first physical activity metric, *Physical Activity %* (*PA*%) is shown in Eq 5. It measures the ratio of *Physical Activity Tweets* compared to the total number of tweets.

$$\begin{array}{c} PA\%=\frac{\left|PA\right|}{\left(|PA|+|NPA|\right)} \end{array}$$ (5)

The guidelines of physical activities from the ACSM and CDC divides activities into two categories which serve as the basis for our second measure. The two categories of activities are: (1) moderately intense activities that burn 3.5 kCals a minute and (2) strenuously intense activities that burn 7.0 kCals a minute. Moderately intense physical activities include yoga, walking and stretching while strenuously intense physical activities include jogging, mountain climbing and aerobics. For a given tweet *T* discussing *M* moderately intense physical activities and *S* strenuously intense physical activities we calculate, *pa*_*weighted*_ in Eq 6. *pa*_*weighted*_ is the *weighted* number of calories burned by participating in all the physical activities discussed in the tweet for one minute.

$$\begin{array}{c} pa_{weighted}\left(T\right)=\left(3.5\times M\right)+\left(7.0\times S\right) \end{array}$$ (6)

### Objectivity and Limitations

All of the measures in Eqs 2–6 make no attempt to take the context of words or the meaning of a tweet into account. While this may limit the ability of our measures to appropriately score tweets containing only a few words, previous researchers have employed this approach and obtained reliable results. Furthermore, by ignoring the context of words we gain a degree of impartiality. We are not the one’s deciding a priori whether a given word, food or activity is associated with obesity. This strategy reduces experimental bias and maintains objectivity.

## Results

### Happiness and Obesity Rate

The first measure we explore is the *happiness* conveyed in individual words from tweets. Mitchell et al. showed that the *happiness* of tweets are correlated with the 2011 obesity rate in MSAs [12]. To validate this result we explore the correlation between the *happiness* of a tweet and the obesity rate of MSAs in our random sample of Twitter data. Recall, our Twitter data contains ∼ 25 million tweets collected during 2012 and 2013 while Mitchell et al.’s data contains ∼ 10 million tweets collected during 2011. Also Mitchell et al. used GHWS obesity rates collected during 2011 while we use obesity rates collected during 2012 and 2013.

**Fig 1:**
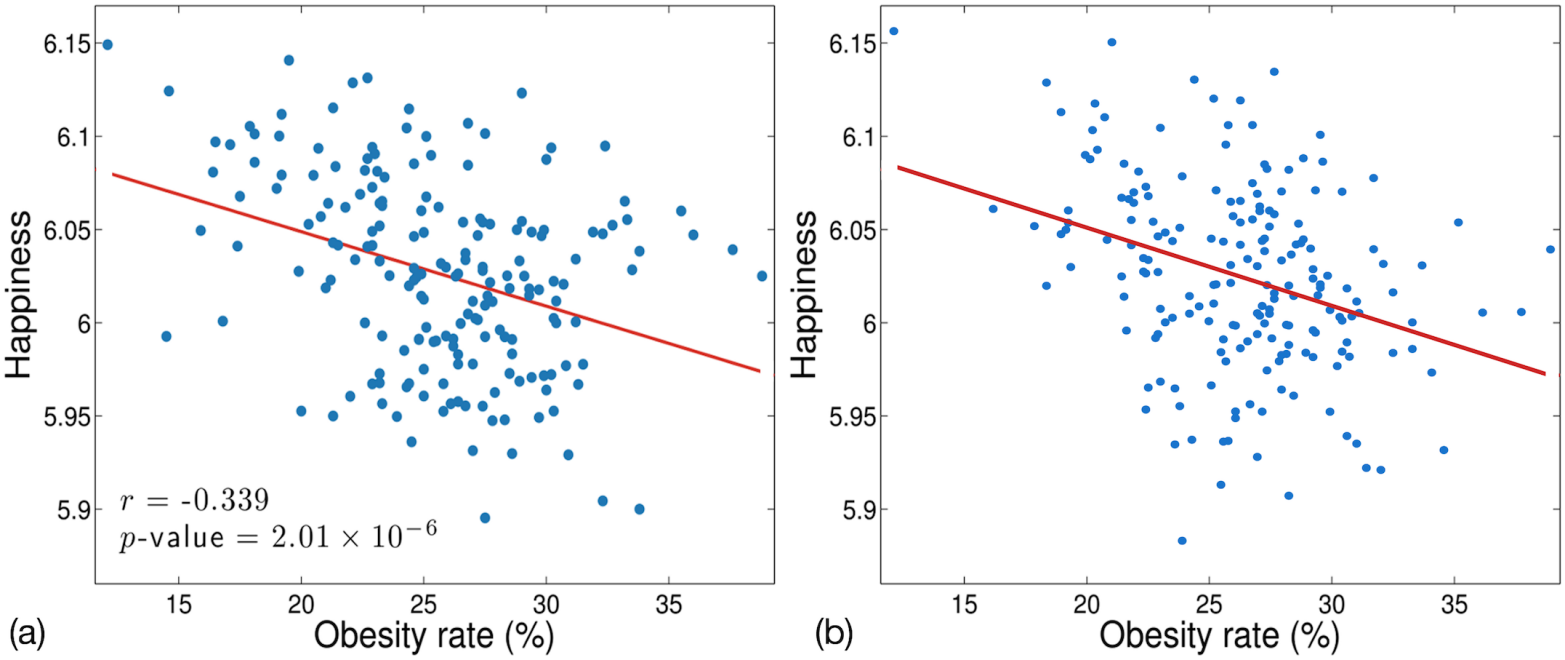
Correlation of *h*_*avg*_ and obesity rate over all MSAs in: (a) 2011 and (b) 2012–2013.

Fig 1 shows the correlation of *h*_*avg*_ and the obesity rate in all the MSAs for: (a) 2011 (Mitchell et al.) and (b) 2012–2013 (our work). The data shows that the happiness people express in tweets generally decreases as the obesity rate increases. This result holds true in 2011 as well as in 2012–2013. Furthermore, the strength of the relationship and the subtleties of the data points are similar. For example, Boulder, CO is the city with the lowest obesity rate and is among the three most happy cities each year. Furthermore Beaumont, TX is in the top 10 MSAs in terms of obesity rate in both data sets and bottom five happiest cities. The Spearman correlation coefficients are similar (*r* = -0.339 in 2011, *r* = -0.318 in 2012–2013) and each have *p*-values far below.001 indicating that the negative correlations are statistically significant. Next, we explore the relationship of five measures of other factors affecting obesity (diet and physical activity) that can be gleamed from Twitter data in a manner similar to the *happiness* metric, *h*_*avg*_.

### Dietary Health and Obesity Rate

Research has shown that diet influences obesity [28, 29]. However, the happiness metric, *h*_*avg*_, does not account for diet. Many foods that are widely considered unhealthy have high happiness values (*h*). For example, the term *cake* has a *h* value = 7.58 Also, healthy foods can have relatively low happiness values. The term *vegan* has a *h* value of 4.82 despite reflecting a diet featuring fruits and vegetables. Furthermore, many healthy and unhealthy foods are not included in the list of terms scored for happiness. As a result, they are completely ignored in the previous analysis.

**Fig 2:**
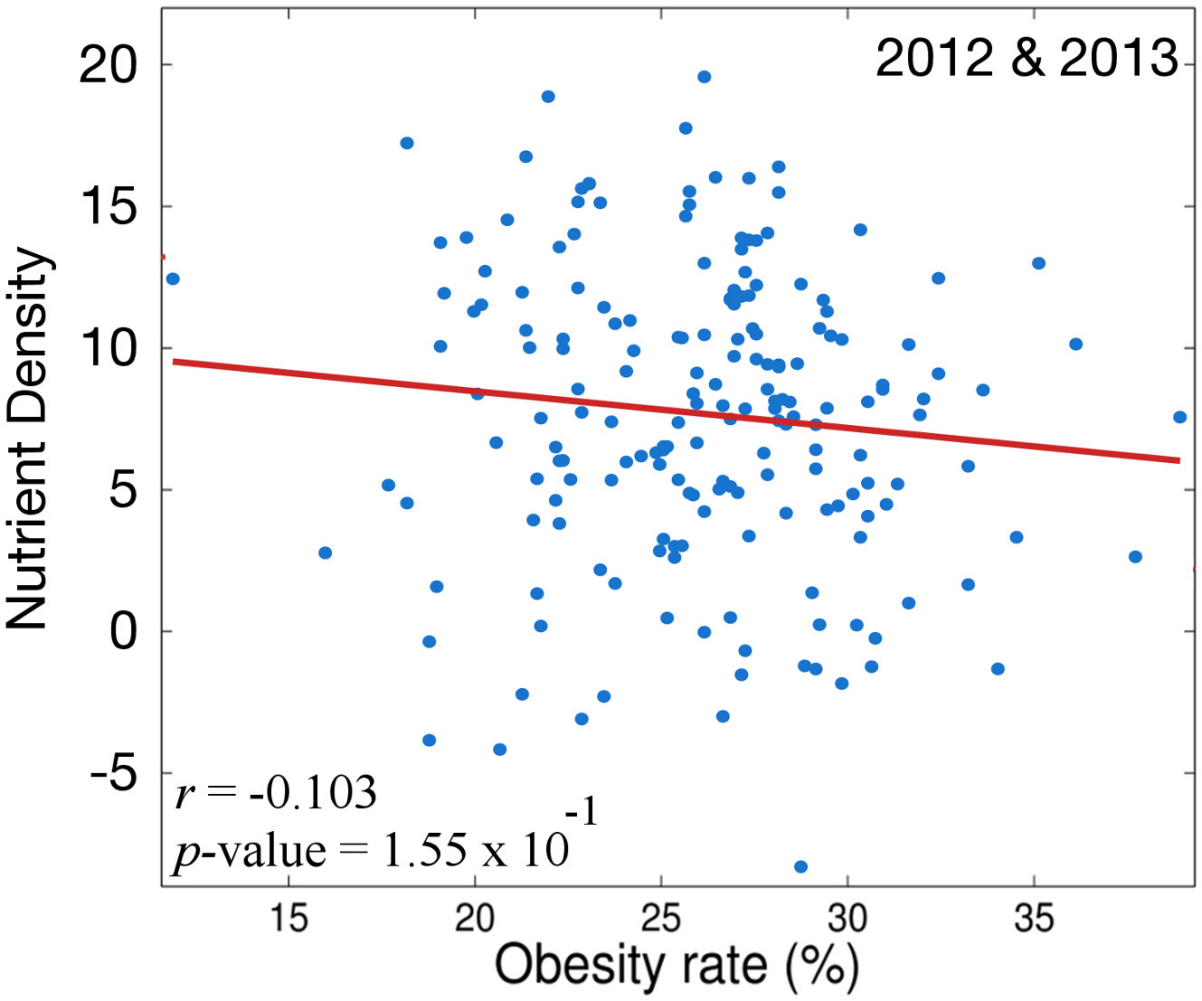
Correlation of *nd*_*avg*_ and BMI over all MSAs for 2012–2013.

To gather insight into the relationship between the foods one tweets about and obesity we explore the correlation between three different measures of the dietary content of a tweet and the obesity rate of MSAs. The first measure we explore is *nd*_*avg*_ shown in Eq 3. Recall, *nd*_*avg*_ reflects the average nutrient density of a tweet. The twitter data we use for this analysis includes more than two million tweets from 2012–2013 mentioning more than six hundred of the 8,000 different foods listed in the USDANDB. The Spearman correlation between *nd*_*avg*_ and obesity rate in all MSAs over 2012–2013 is shown in Fig 2.

Fig 2 shows that there is not a statistically significant relationship between the nutrient density of the foods people discuss in their tweets and obesity rate. This result is unexpected. Given our previous result related to the happiness of tweets and the established relationship between diet and obesity, we anticipated a statistically significant negative correlation. We pursue an explanation by identifying the ten foods that are most strongly negatively and positively correlated with obesity. These results are shown in Table 1.

**Table 1:** Top Ten Foods Most Negatively & Positively Correlated With Obesity Rate.

*Negative*				*Positive*			
Food	*r*	*p*-value	NRF	Food	*r*	*p*-value	NRF
wine	-.407	*p* <.001	10.0	chicken nuggets	.207	*p* <.01	5.9
coffee	-.372	*p* <.001	4.5	ham	.174	*p* <.01	-6.4
banana	-.325	*p* <.001	51.8	french fries	.165	*p* <.05	-15.2
espresso	-.314	*p* <.001	3.8	chicken wings	.145	*p* <.05	6.8
croissant	-.285	*p* <.001	-9.1	sausage	.129	p >.05	-19.3
apple	-.282	*p* <.001	46.7	biscuit	.113	p >.05	0.2
salmon	-.274	*p* <.001	36.0	collards	.097	p >.05	392.5
quinoa	-.268	*p* <.001	31.8	bbq sauce	.092	p >.05	-2.5
brie	-.265	*p* <.001	-8.5	fried chicken	.088	p >.05	8.9
macaroon	-.261	*p* <.001	-8.4	gravy	.084	p >.05	-4.2

Table 1 elucidates several insights into the set of tweets that discuss food. The first is that areas with lower obesity rates do not exclusively discuss foods that are nutritionally dense. Similarly areas with high obesity rates discuss a mix of nutritionally dense and non-nutritionally dense foods. Specifically, both lists contain multiple foods with positive and negative NRF Index values and the food with the highest NRF Index value (collards) is correlated with high obesity rates.

It is important to note that our nutrient density metric ignores the quantity and preparation of the food consumed in the tweet. These limitations could explain the lack of a significant relationship between the nutrient density of foods people discuss in tweets and their obesity rate. However, the correlation coefficients and *p*-values in Table 1 reveal that tweets that discuss food, regardless of their nutritional density, are more likely to be negatively correlated with obesity rate than positively correlated. The absolute value of the correlation coefficient of the food tenth most negatively correlated with obesity is ∼ 25% larger than the absolute value of the correlation coefficient for the food most positively correlated with obesity rate. The *p*-values in Table 1 also reflect this trend. The relationship between all the foods negatively correlated with obesity rate are statistically significant (*p* <.05) while only the top four foods positively correlated with obesity rate are statistically significant.

**Fig 3:**
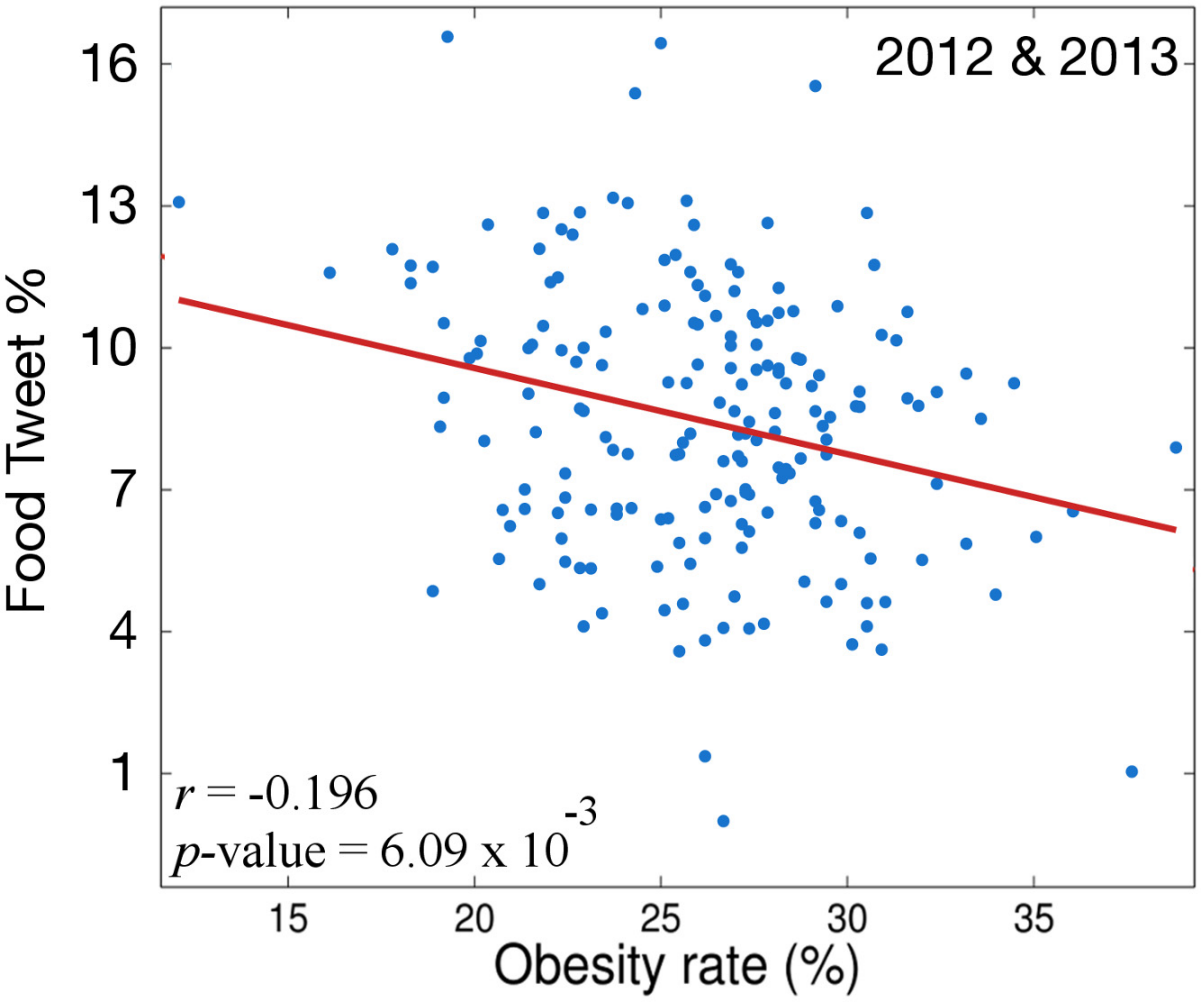
Correlation of *FT*% and BMI over all MSAs for 2012 & 2013.

Given these two observations we explore the data to see if the frequency with which individuals tweet about food, regardless of its nutritional density, is correlated with obesity. We use the same twitter data as our previous analysis. However, in this version we measure the ratio of *Food Tweets* compared to the total number of tweets. This metric, *FT*% is shown in Eq 2. The Spearman correlation between *FT*% and obesity over all MSAs for each is shown in Fig 3.

Fig 3 shows that the frequency with which people discuss foods in tweets generally decreases as obesity rate increases. For example, San Francisco, CA is the MSA with one of the highest *FT*% and is among the ten MSAs with the lowest obesity rate. Similarly, several of the MSAs with top twenty obesity levels (Flint, MI; Mobile, AL; Rockford, IL) are amongst the bottom twenty MSAs in terms of *FT*%. However, the negative correlation between *FT*% and obesity rate is not as strong as the negative correlation between *h*_*avg*_ and obesity rate. Furthermore, the negative correlation between *FT*% and obesity is not immediately obvious. There is not a quorum of established evidence that shows that the more people discuss food the less obese they are.

**Fig 4:**
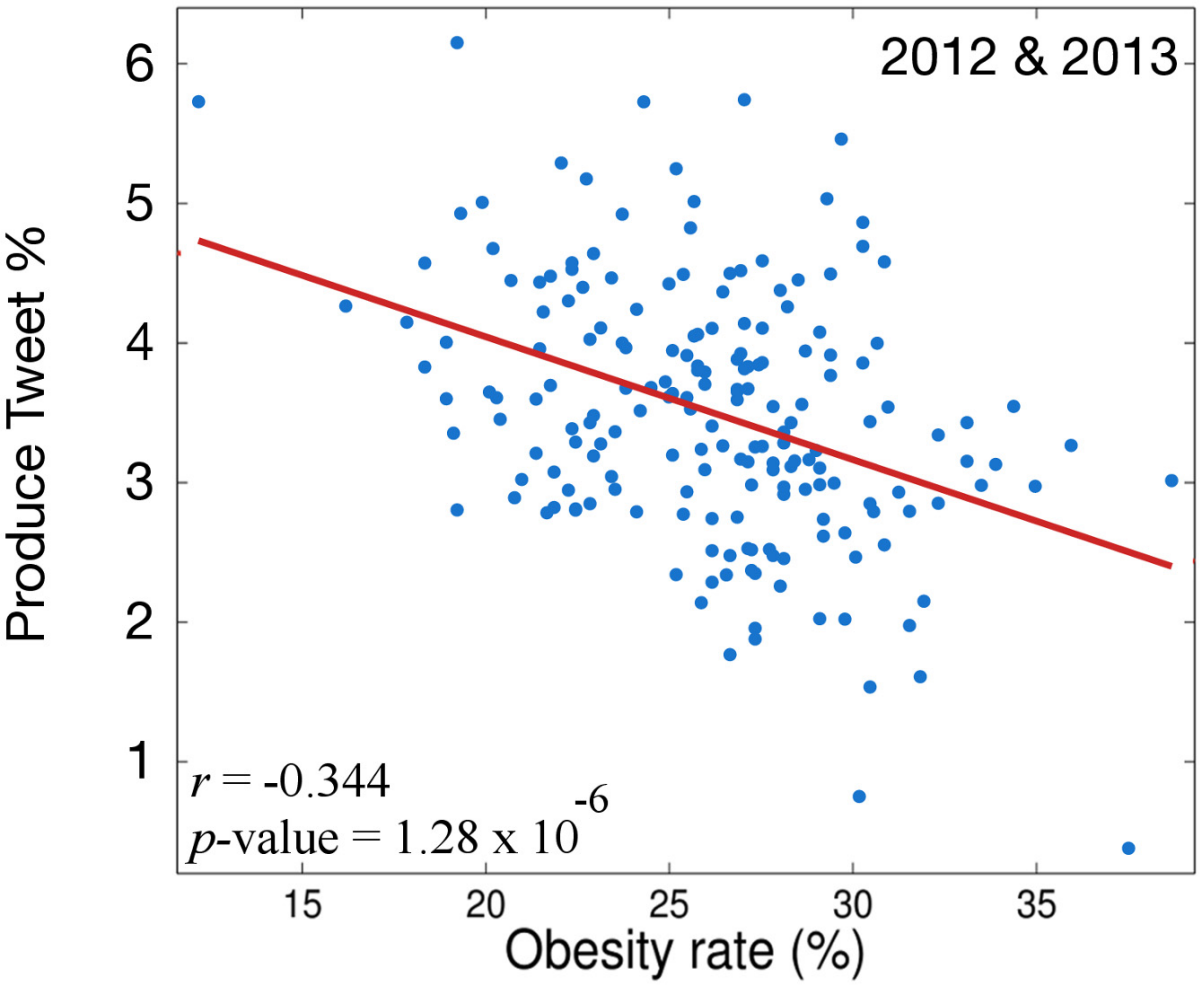
Correlation of *Prod*% and obesity over all MSAs for 2012 & 2013.

In order to examine our data further we explore our final measure of the diet of a MSA: *Produce %* (*Prod*%). Recall, *Prod*% marries together the nutritional aspects of *nd*_*avg*_ with the coarse granularity of *FT*%. It reflects the percentage of total tweets that discuss at least one of the foods listed in either the *Fruits and Fruit Juices* or *Vegetable and Vegetable Products* sections of the USDANDB. The twitter data we use for this analysis includes more than one million tweets from 2012–2013 mentioning more than 150 different fruits, vegetables or fruit/vegetable related products. The Spearman correlation between *Prod*% and obesity rate over all MSAs is shown in Fig 4.

Fig 4 shows that the *Prod*% metric reconciles the trends we saw in our previous explorations with the measures *nd*_*avg*_ and *FT*%. The frequency with which people tweet about fruits, vegetables or related products increases as obesity decreases.

Intuitively this makes sense. Fruits and vegetables are some of the highest scoring items on the NRF Index, so eating them regularly should decrease the obesity rate. The previous measure, *nd*_*avg*_, attempted to account for this but over penalized tweeters for mentioning average and below average foods on the NRF Index. The *FT*% metric offered a much coarser level of granularity but did not consider the nutritional density of the foods being discussed in a tweet at all. By including nutritional density at a coarse level of granularity we are able to reveal a correlation with obesity rate (*r* = -0.344) that is similar in magnitude to the correlation between *h*_*avg*_ and obesity rate. Next, we investigate the discussion of physical activity levels on Twitter and their relationship to the obesity rate in MSAs.

### Physical Activity Level and Obesity Rate

Along with happiness and diet, research has shown that the physical activity level of individuals affects obesity [21–23]. However, none of our previously explored measures (*h*_*avg*_, *nd*_*avg*_, *FT*% and *Prod*%) account for discussions of physical activities within tweets. As a result, we explore two different measures of discussions of physical activity within our Twitter data set.

**Fig 5:**
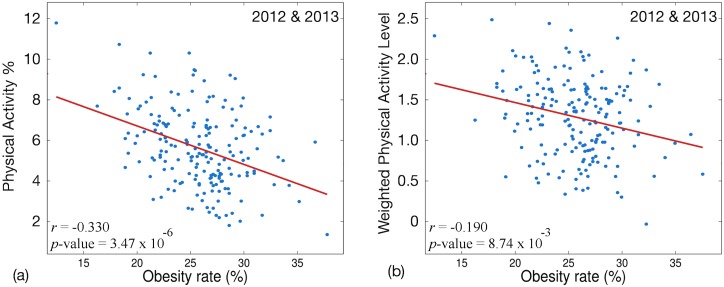
Correlation of obesity rate and (a) *PA*% and (b) *pa*_*weighted*_ over all MSAs in 2012 & 2013.

Our first physical activity measure, *Physical Activity %* (*PA*%) measures the ratio of Physical Activity related tweets compared to the total number of tweets. Our second measure weights physical activities according to the intensity levels published by guidelines of the ACSM and CDC. These two measures are shown in Eqs 5 and 6. The Spearman correlation between *PA*% and obesity rate and *pa*_*weighted*_ and obesity rate in all MSAs over 2012 and 2013 is shown in Fig 5(a) and 5(b).

The twitter data we use for this analysis includes more than three million tweets from 2012 and 2013 mentioning more than eighty of the physical activities listed by the ACSM and CDC. Almost two million tweets discuss forty-eight different activities of moderate intensity and more than one million tweets discuss thirty-six different activities of strenuous intensity.

The *pa*_*weighted*_ values of the tweets in our data set vary. The minimum is zero, which reflects a tweet that does not discuss any physical activities from the list published by the ACSM and CDC. The maximum *pa*_*weighted*_ observed in our data set is 24.5. However, over 99% of the tweets in our data set have *pa*_*weighted*_ values of either: 0, 3.5 or 7.

Fig 5 shows that there is a statistically significant negative correlation between both *PA*% and *pa*_*weighted*_ and the obesity rate in MSAs. However, the relationship between *PA*% and obesity rate is stronger (*r* = -0.330) than the relationship between *pa*_*weighted*_ (*r* = -0.190) and obesity rate. This result may seem unexpected. The *pa*_*weighted*_ metric offers the capability to combine the calories burned from multiple activities based on their intensity level. Given these additional capabilities one might expect it to correlate better with obesity rate than the basic *PA*% metric. To gather additional insight we calculate the activities most positively and negatively correlated with obesity rate in Table 2. Table 2 only includes five activities in each column because there are so few physical activities that have a positive statistically significant correlation with obesity rate.

**Table 2:** Top Five Physical Activities Most Negatively & Positively Correlated With Obesity Rate.

*Negative*				*Positive*			
Activity	*r*	*p*-value	Intensity	Activity	*r*	*p*-value	Intensity
golf	-.327	*p* <.001	moderate	basketball	.218	*p* <.01	strenuous
yoga	-.318	*p* <.001	moderate	hunting	.182	*p* <.01	moderate
hiking	-.273	*p* <.001	moderate	football	.176	*p* <.05	strenuous
racquetball	-.246	*p* <.001	strenuous	dancing	.151	p >.05	moderate
lacrosse	-.222	*p* <.01	strenuous	coaching	.128	p >.05	moderate

Table 2 shows that areas with low obesity and areas with high obesity engage in twitter discussions of a mixture of moderately and strenuously intense activities. Both lists include three moderately intense activities and two strenuously intense activities. However, Table 2 also shows that areas with lower obesity rates simply tweet more about physical activities than areas with high obesity rates. The absolute value of the correlation coefficient for the fifth most negatively correlated activity is higher than the absolute value of the correlation coefficient for the activity most positively correlated with obesity.

It is important to note that our physical activity measures ignore if an individual’s discussion of an activity reflects them physically engaging in it or merely witnessing it in some manner. The inability to make this distinction could explain the lack of a more significant relationship between the intensity levels of physical activities and obesity rate.

However, these insights do reveal similarities between the measures: (1) *nd*_*avg*_ and *Prod*% and (2) *pa*_*weighted*_ and *PA*%. In both cases adding too much detail to the measure derived from tweets diluted the relationship between the quantities of interest. This is a valuable lesson learned. Given the complexity of Mitchell et. al.’s happiness metric, *h*_*avg*_, we assumed we would need measures of discussions of food and physical activities with a similar structure. However, this is not the case. The more coarse measures *Prod*% and *PA*% had a stronger relationship to obesity rate than the nuanced measures *nd*_*avg*_ and *pa*_*weighted*_. Next, we explore the extent to which these measures provide different insight about the obesity rate of a MSA and evaluate the extent to which each correlates with a a MSA-level survey measure of a similar variable.

### Evaluation of Measures

The results we have presented thus far demonstrate that three measures (*h*_*avg*_, *Prod*% and *PA*%) which can be obtained from geo-tagged tweets have a statistically significant negative correlation with the obesity rate of a MSA and that correlation is on the order of -0.30. However, we have not presented any results which show that: (1) the three measures (*h*_*avg*_, *Prod*% and *PA*%) have unique relationships with the obesity rate of a MSA and (2) the measures actually quantify the happiness, diet and physical activity level of a MSA.

We address both of these questions by computing the correlation among seven variables. Three of the seven variables are the measures of happiness, diet, and physical activity that can be gleamed from Twitter discussions within a MSA and are most correlated with obesity rate: *h*_*avg*_, *Prod*% and *PA*%. The other four variables reflect MSA-level data collected by the GHWS survey data. These variables are the: (1) obesity rate of a MSA, (2) percentage of individuals in a MSA who report that they eat a healthy diet, (3) percentage of individuals in a MSA who report that they exercise frequently and (4) Well-Being Index of a MSA. The Well-Being Index is computed by aggregating the responses from participants to five statements. Each participant rates their agreement with each statement on a 0 (very strong disagreement) -10 (very strong agreement) scale. The statements are [7]:

1. I am satisfied with my present life situation and anticipated life situation.
2. My daily feelings and mental state are healthy.
3. I have the physical ability to live a full life.
4. The behaviors I engage in positively affect my physical health.
5. Within my community I feel safe, satisfied and optimistic.

**Fig 6:**
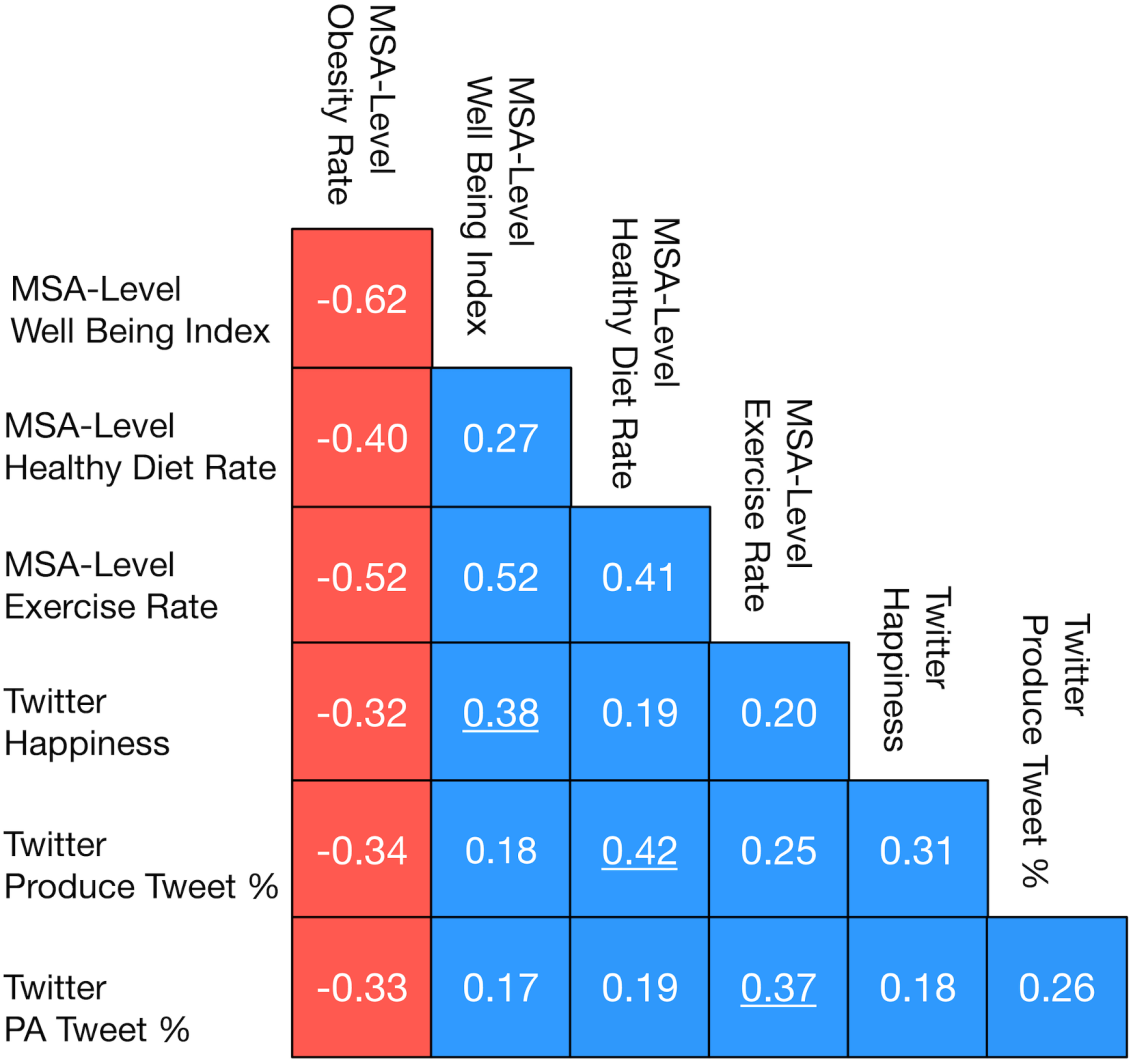
Correlation among four MSA-level measures and three Twitter measures of MSAs. Blue boxes reflect a positive correlation, red boxes reflect a negative correlation.

Fig 6 visualizes the lower triangle of a matrix of the Spearman correlations among the seven variables. The blue boxes in Fig 6 reflect a positive correlation, red boxes reflect a negative correlation. This data shows that each of the measures we computed from Twitter discussions within a MSA (*h*_*avg*_, *Prod*% and *PA*%) are more correlated with the obesity rate of a MSA than they are correlated with any of the other measures computed from Twitter data. This provides evidence that each of the three measures reflect different factors which are correlated with the obesity rate of a MSA. In other words, these three measures are not simply different methods of quantifying the same variable.

Furthermore, each of the three measures gleamed from our Twitter corpus is more correlated with the MSA-level measure of a similar variable from GHWS than any other variable. To help elucidate this trend we have underlined the correlation coefficient of the variables with the strongest correlation to *happiness*, *Prod*% and *PA*%. While, this trend does not completely rule out the existence of confounders within our Twitter-level measures, it provides evidence that *h*_*avg*_, *Prod*% and *PA*% are actually reflecting the level of happiness/well-being, diet/healthy-eating and physical activity/exercise within a MSA as opposed to three completely unrelated variables. Next, we review related work, discuss the validity and limitations of our results and provide directions for future work.

## Discussion

We are not the first researchers to explore modeling human behavior with content from Twitter. Emotions have been accurately captured at different levels of granularity from tweets by using hashtags [30] and sentiment analysis [31, 32]. Given these classification capabilities other researchers have used Twitter data to explore the emotional states individuals go through in a 24 hour period [33] and while watching sporting events [34].

Tweets have also been used to model consumer confidence [35] and identify major news events that cause breaking points in public opinion [36]. They have served as a platform to explore the unique characteristics of astrophysicists [37] and been analyzed to characterize varieties of the Spanish dialect on a global scale [38]. However, the two studies most related to our research are Broniatowski et al.’s work on modeling the spread of influenza through tweets [39] and Mitchell et al.’s exploration of the relationship between the happiness of a tweet and its geographic origin [12].

Since we have already reviewed and validated Mitchell et al.’s work, we only focus on Broniatowski et al. here. Broniatowski et al identified measures that distinguish tweets relevant to influenza from other tweets. In this paper we adopt this strategy to identify measures related to the variation in obesity rate of MSAs from 2012–2013.

We have identified three measures which can be gleamed from Twitter content related to happiness, diet and physical activities. Each of these measures has a statistically significant negative correlation with obesity on the order of -0.30. Furthermore, we have provided evidence that these measures reflect different variables associated with obesity and that these variables actually reflect the happiness, diet and physical activity levels of MSAs. Ultimately, this work has furthered the research effort in understanding obesity by providing a new path through social media data for the development of population-scale measures of factors related to obesity.

Despite these results, internal and external validity threats affect our study. Threats to internal validity arise when factors affect the dependent variables without the evaluators’ knowledge. It is possible that some flaws in the implementation of our metrics could have affected the results of the evaluation. However, the algorithms we used to compute the metrics passed several internal code reviews and the strength of the relationship between our implementation of the happiness metric, *h*_*avg*_, and the obesity rate in MSAs is similar to previously published results [12]. Threats to external validity occur when the results of the evaluation cannot be generalized. Although the evaluation was performed for two years of data over 189 MSAs the results cannot be generalized to: (1) other urban areas, (2) during different years or (3) different Twitter data sets.

Furthermore, there are issues that must be addressed with how well a geo-tagged Twitter data set can represent the obesity rate of a population. Only 15% of online adults regularly use Twitter, and 18–29 year-olds and minorities tend to be more highly represented on Twitter than in the general population [40]. Furthermore, on Twitter, 95% of users never geo-tag a single tweet and only ∼ 1% of users geo-tag the majority of the tweets they post. Also, the extent to which the individual ‘tweeter’ is represented in our Twitter corpus is biased. Very passive users (< 50 tweets per year) and very active users (> 1000 tweets per year) geo-tag a smaller percentage of tweets than moderate users (50–1000 tweets per year) [40]. Finally, we collected only a random sample of all tweets during 2012–2013. Ultimately, these limitations mean that the data set which informed our study is a non-uniform subsample of statements made by a non-representative portion of MSA populations.

Even with these limitations and validity threats we have only scratched the surface of what is possible using social media datasets. In particular, Tables 1 and 2 could be very illuminating. One can observe that the top foods and physical activities positively (espresso, yoga) and negatively (french fries, hunting) correlated with obesity rate may have social and cultural underpinnings (i.e. income and education levels).

This would not be unexpected. Recall, previous work showed that the happiness of a MSA, which correlates with our diet and physical activities measures, has statistically significant positive correlations with: (a) the percentage of households with median income levels and (b) the percentage of the individuals living in an area who have obtained a bachelor’s degree. Also, happiness has a statistically significant negative correlation with families living below the poverty line. In future work, we plan to use the census data for 2012 to investigate how different demographics across urban areas are correlated with our measures of diet (*Prod*%) and physical activity level (*PA*%).

Additionally, we have not examined whether or not these methods have any predictive power. Future work will look at how observed changes in the measures which can be gleamed from Twitter data, predict changes in the obesity rate of MSAs. We plan to pursue this in future work using content from Twitter and the GHWS data collected in 2014 and 2015.

## Supporting Information

S1 Dataset Dataset for *h*_*avg*_ over all MSAs for 2012 & 2013. (CSV)

S2 Dataset Dataset for *nd*_*avg*_ over all MSAs for 2012 & 2013. (CSV)

S3 Dataset Dataset for *FT*% over all MSAs for 2012 & 2013. (CSV)

S4 Dataset Dataset for *Prod*% over all MSAs for 2012 & 2013. (CSV)

S5 Dataset Dataset for *pa*_*weighted*_ over all MSAs in 2012 & 2013. (CSV)

S6 Dataset Dataset for *PA*% over all MSAs in 2012 & 2013. (CSV)

S1 Table Foods Most Negatively Correlated With Obesity Rate. (CSV)

S2 Table Foods Most Positively Correlated With Obesity Rate. (CSV)

S3 Table Physical Activities Most Negatively Correlated With Obesity Rate. (CSV)

S4 Table Physical Activities Most Positively Correlated With Obesity Rate. (CSV)
